# Social behavioral testing and brain magnetic resonance imaging in chicks exposed to mobile phone radiation during development

**DOI:** 10.1186/s12868-016-0266-7

**Published:** 2016-06-10

**Authors:** Zien Zhou, Jiehui Shan, Jinyan Zu, Zengai Chen, Weiwei Ma, Lei Li, Jianrong Xu

**Affiliations:** Department of Radiology, Ren Ji Hospital, School of Medicine, Shanghai Jiao Tong University, 160 Pujian Road, Shanghai, 200127 China; Department of Geriatrics, South Campus, Ren Ji Hospital, School of Medicine, Shanghai Jiao Tong University, 2000 Jiangyue Road, Shanghai, China

**Keywords:** Chick embryo, Mobile phone radiation, Social behaviors, MRI

## Abstract

**Background:**

The potential adverse effect of mobile phone radiation is currently an area of great concern in the field of public health. In the present study, we aimed to investigate the effect of mobile phone radiation (900 MHz radiofrequency) during hatching on postnatal social behaviors in chicks, as well as the effect on brain size and structural maturity estimated using 3.0 T magnetic resonance imaging. At day 4 of incubation, 76 normally developing chick embryos were divided into the control group (n = 39) and the radiation group (n = 37). Eggs in the radiation group were exposed to mobile phone radiation for 10 h each day from day 4 to 19 of incubation. Behavioral tests were performed 4 days after hatching. T2-weighted MR imaging and diffusion tensor imaging (DTI) were subsequently performed. The size of different brain subdivisions (telencephalon, optic lobe, brain stem, and cerebellum) and corresponding DTI parameters were measured. The Chi-square test and the student’s *t* test were used for statistical analysis. P < 0.05 was considered statistically significant.

**Results:**

Compared with controls, chicks in the radiation group showed significantly slower aggregation responses (14.87 ± 10.06 vs. 7.48 ± 4.31 s, respectively; P < 0.05), lower belongingness (23.71 ± 8.72 vs. 11.45 ± 6.53 s, respectively; P < 0.05), and weaker vocalization (53.23 ± 8.60 vs. 60.01 ± 10.45 dB/30 s, respectively; P < 0.05). No significant differences were found between the radiation and control group for brain size and structural maturity, except for cerebellum size, which was significantly smaller in the radiation group (28.40 ± 1.95 vs. 29.95 ± 1.41 cm^2^, P < 0.05). The hatching and heteroplasia rates were also calculated and no significant difference was found between the two groups.

**Conclusions:**

Mobile phone radiation exposure during chick embryogenesis impaired social behaviors after hatching and possibly induced cerebellar retardation. This indicates potential adverse effects of mobile phone radiation on brain development.

## Background

With the development of communication technology, the use of mobile phones has steadily increased worldwide. Statistics from the International Telecommunications Union (ITU) show that there were more than 7.0 billion mobile-cellular subscriptions by the end of 2015 [1], which is almost equal to the world population. In recent years, increasing attention has been paid to the potential adverse effects of mobile phone radiation on human health, such as its influence on sperm quality, and the increase in the incidence of brain tumors, sleep disorder, and mental problems [2–7]. Global system for mobile communication (GSM) is widely used in mobile phones and its electromagnetic waves range in frequency from 300 MHz to 3 GHz. The electromagnetic field (EMF) produced by these electromagnetic waves could lead to various pathological changes in viable tissues and cells via heat-related and non-heat-related effects, such as oxidative stress, Ca^2+^-signal channel’s adjustment, and DNA damage [8–11].

Among the various health concerns related to mobile phones, the question of whether using a mobile phone frequently during the gestation period will have an adverse effect on fetal growth is a common concern among pregnant women. Many pregnant women wear so-called radiation-proof clothes to avoid potential influence of EMF on the fetus. One epidemiological research study showed that exposure to mobile phones prenatally and postnatally is associated with emotional problems and hyperactivity in children at approximately the age of school entry [12]. However, another epidemiological study suggests that maternal mobile phone use during pregnancy does not increase the risk of behavioral problems in children [13]. Few clinical studies have been performed because of ethical limitations. Some experiments in small animals such as rats and chicks have been performed to investigate the effect of EMF on brain development, with conflicting results [14–17].

The chick embryo is an accessible and economical model, which has an extensive history of use in developmental biology, transplantation research, pharmaceutical teratogenicity evaluation, and cancer research [18]. EMF’s effects on chick embryonic development are mainly investigated with respect to embryo survival and hatchability, macro-shape or microstructural changes in histology, and oxidative stress levels in tissues [16, 19, 20]. To our knowledge, the effect of EMF exposure during development on the social behaviors of chicks after hatching has not been investigated to date. Chicks’ social behaviors, which can be monitored to assess brain development, are evaluated using a “social-separation-stress test” (SSST) model [21]. The SSST is designed to evaluate chicks’ willingness to form groups (evaluating belongingness and aggregation) and to communicate with each other by vocalization. The SSST has been used to examine quantitatively the effects of drug exposure on chick development [21, 22]. Magnetic resonance imaging (MRI) is a powerful, noninvasive tool used in brain developmental research because of its high spatial and tissue-contrast resolution without interference from the skull, flexible imaging plane orientation, provision of functional information, and a lack of radialization. Diffusion tensor imaging (DTI) can be used to reflect the structural character and maturation of the brain by measuring DTI parameters [apparent diffusion coefficient (ADC) and fractional anisotropy (FA) value] [23]. The present study had two primary goals. One was to investigate the effect of EMF exposure during development on postnatal social behaviors (aggregation behavior, belongingness and vocalization tests) in chicks. The second was to investigate the effect of EMF on brain size and structural maturity of different brain subdivisions (telencephalon, cerebellum, optic lobe and brain stem) after hatching using MRI.

## Methods

### Chick embryos and mobile phone radiation

The experiment was approved by the Institutional Animal Care and Use Committee of Shanghai Jiao Tong University School of Medicine. The experiment was carried out on three successive batches of eggs. In each batch, thirty Hy-line White eggs, each weighing 50–55 g, were obtained from a commercial hatchery (Shanghai Qigan poultry factory, China) and placed in an incubator with automatic temperature (37.8 °C) and humidity (60 %) control. Eggs were automatically rolled every 2 h. After 4 days of incubation, eggs were candled with a hand-held light source to observe whether they were developing normally. We considered the chick embryo to be developing normally if the capillary network was observed. Undeveloped and unfertilized eggs were removed from the incubator at this point. The remaining eggs were randomly divided into a radiation group and a control group. The number of normally developing eggs at day 4 of incubation, and the number of eggs in the EMF radiation and control groups in each experimental batch are given in Table 1.

**Table 1:** The number of eggs for hatching, the number of normally developing eggs at day 4 of incubation, and the number of eggs randomized into radiation group and control group, in each batch of the experiment

Experimental batch no.	No. of eggs for hatching	No. of normally developing eggs at day 4 of incubation	No. of eggs assigned to the radiation group (n = 37)	No. of eggs assigned to the control group (n = 39)
1	30	26	13	13
2	30	25	12	13
3	30	25	12	13

**Fig. 1:**
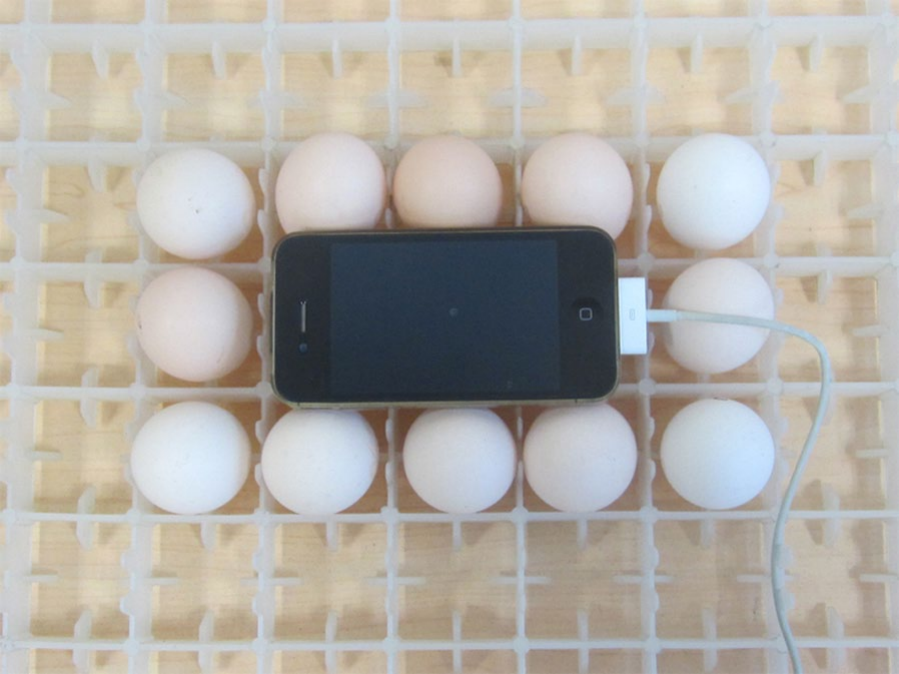
An iPhone 4s (GSM 900 MHz) is placed in the center of the hatching plate as the radiation source for the experimental group

**Fig. 2:**
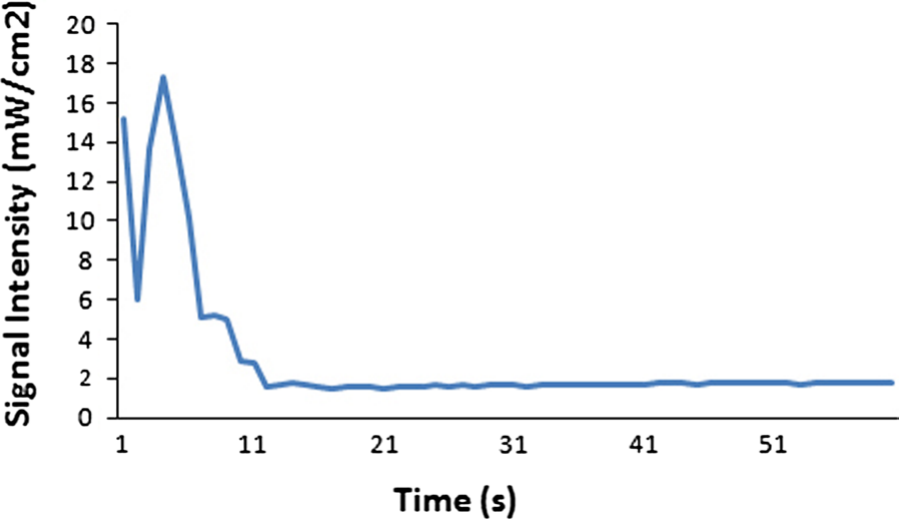
The signal intensity of electromagnetic radiation next to the iPhone 4s while it rings for 1 min

Eggs in the radiation group were moved into another incubator in a neighboring room. The distance between the two incubators was more than 10 m, to prevent any influence of the mobile phone radiation on the eggs in the control group. An iPhone 4s (GSM 900 MHz) was used as a source of radiation and placed in the center of the hatching plate. Experimental eggs were placed around the mobile phone as shown in Fig. 1. The average intensity of electromagnetic radiation next to the iPhone 4s during 1 min of ringing was 3.03 µW/cm^2^, as measured by an electromagnetic radiation meter (TM1390, Shenzhen TECMAN electronics Co., Ltd., Shenzhen, China). Figure 2 shows the signal intensity of the radiation emitted by the iPhone 4s while it was ringing. Embryos in the experimental group were continuously irradiated for 10 h each day (from 8 a.m. to 6 p.m.) from day 4 to 19 of incubation. An Android-based custom-made software was used to call the iPhone 4s in the incubator automatically. Each call lasted 1 min with 30 s gaps between calls. A USB cable was connected to the iPhone 4s for the entire duration of the experimental to keep the battery charged. The surface temperature of the experimental eggs was 37–38 °C, which was randomly measured five times a day to exclude possible heating effects of electromagnetic radiation and/or battery charging. At day 19 of incubation, eggs were placed in the hatcher tray for hatching.

### Examination of hatching and social behaviors

From day 20 to 23 of incubation, hatching was counted every day in the radiation and control group. Hatched chicks with heteroplasia, such as cyclopia, inability to stand and abnormal locomotion, were examined. The hatching rate and heteroplasia rate were calculated and statistically analyzed. The hatching rate was calculated as the number of hatched chicks in a group divided by the number of eggs viable at day 4 of incubation. The heteroplasia rate was calculated as the number of hatched chicks with heteroplasia in a group divided by the number of eggs viable at day 4. Unhatched eggs were cracked and checked at day 25 of incubation.

Chick embryos usually hatch after 20 or 21 days of incubation. We defined day 20 of incubation as post-hatching day 0. Behavioral tests were performed on post-hatching day 4 at room temperature (25–26 °C). The procedures were adapted from previously published work [22] and are described below.

#### Aggregation behavior test

**Fig. 3:**
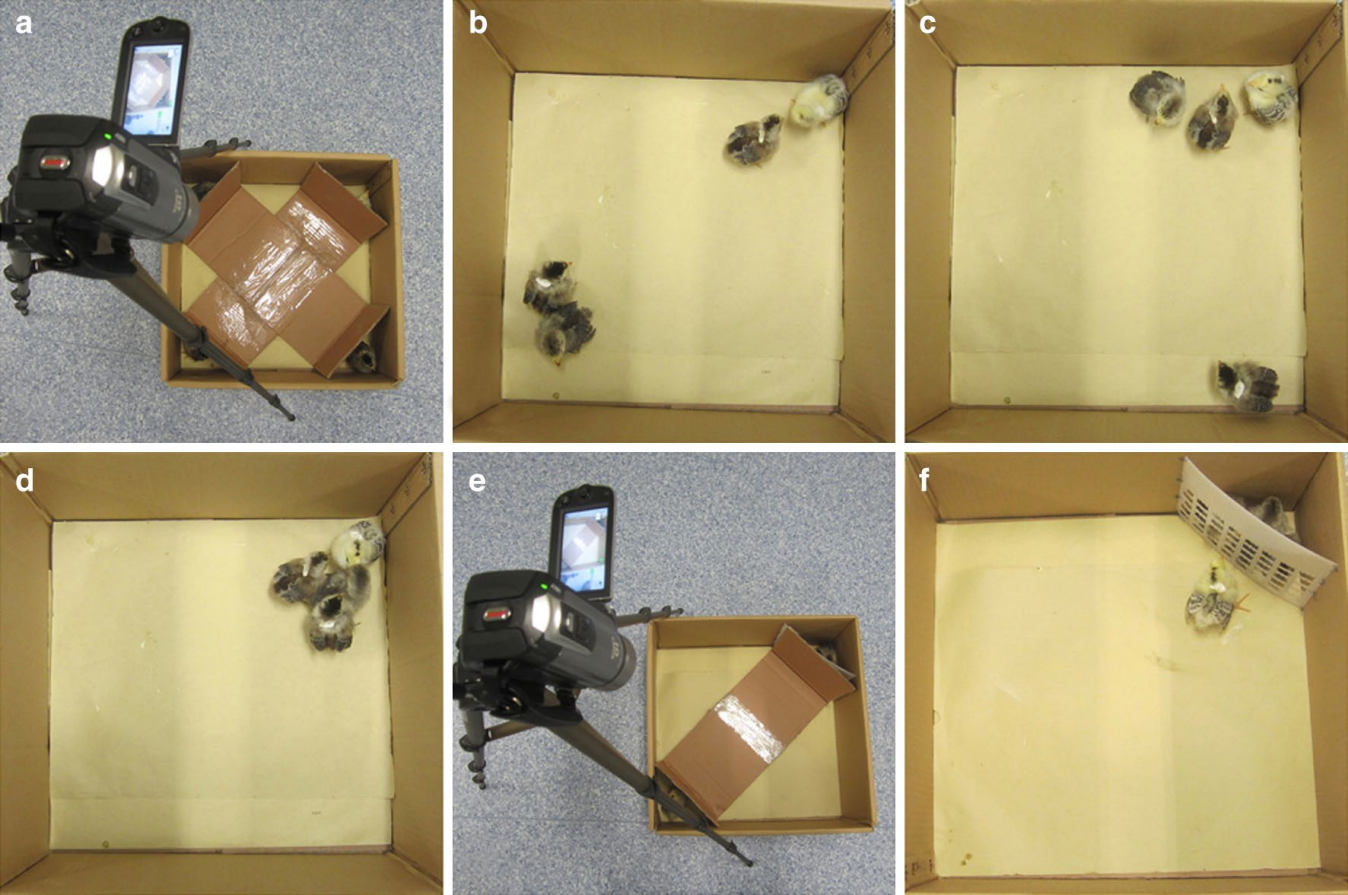
The aggregation behavior test and belongingness test. **a** Test apparatus of the aggregation behavior test; **b** the time point at which the two chicks are aggregated in the aggregation behavior test; **c** the time point at which three chicks are aggregated; **d** the time point at which four chicks are aggregated; **e** test apparatus of the belongingness test; **f** the time point at which the isolated chick reaches the mesh fence at the opposite corner

As shown in Fig. 3a, a cardboard box of 45 × 45 × 24 cm (L × W × H) was used as the apparatus. Its floor was covered with a paper towel and four cardboard fences were placed at four corners to create triangular spaces (18 × 18 × 25 cm). A video camera was positioned over the box for recording. Four of the chicks in the experimental or control group were randomly chosen, and placed in four isolated corners with fences separating them. After the fences were removed simultaneously, aggregation behavior of these four chicks was recorded using the camera. The time required for two, three, and all four chicks to aggregate was noted as shown in Fig. 3b–d. Three such tests were carried out in each of the experimental and control groups, and the average aggregation time was calculated.

#### Belongingness test

The apparatus for the belongingness test is shown in Fig. 3e. It consisted of a cardboard box of the same dimensions as the apparatus used in the aggregation behavior test. Its floor was covered with a paper towel and one cardboard fence was placed at one corner to create a triangular space (18 × 18 × 25 cm). A plastic mesh fence was placed at the opposite corner to create a space of the same size. A video camera was positioned over the box for recording. Another cardboard box of the same size but without the plastic mesh fence was used as an open-field area to be used for acclimation before performing the test. Four chicks in the experimental or control group were placed in the open field for 1 min. Then, three of them were transferred to the corner of the apparatus that was separated by the mesh fence. The remaining chick was put in the opposite corner, behind the cardboard fence. After 30 s, the cardboard fence was removed and the isolated chick could move freely. The time to reach the mesh fence at the opposite corner was recorded as shown in Fig. 3f. Three tests were carried out for each chick and the average arrival time was calculated.

#### Vocalization test

The apparatus for vocalization test was the same as that for the belongingness test. A quiet room was used for this test. Four chicks in the experimental or control group were placed in an open field for 2 min. Then, one of them was transferred to the corner of the apparatus that was separated by the cardboard fence. The chick’s tweets were recorded for 30 s using a sound meter (TM810 M, Shenzhen TECMAN electronics Co., Ltd., Shenzhen, China). The distance between the sound meter and the apparatus was 2 m. Three tests were carried out for each chick and the average sound level in decibels was calculated.

### MR imaging and measurement

After the behavioral tests, all chicks were anesthetized by an abdominal injection of 0.15 ml 5 % chloral hydrate before imaging. MRI scans were performed using a 3.0 T GE Signa Excite System (GE Medical Systems, Waukesha, WI, USA) with a four-channel dedicated animal coil. The inner diameter of the coil was 4 cm. The head of a completely anaesthetized chick was placed in the center of the coil. A plastic support was used to fix the chick’s head such that the chick lay on its back. Anatomical imaging of sagittal brain slices was performed using the 2-dimensional T2-weighted fast spin-echo (FSE) sequence: TR/TE 4800/91 ms, FOV 4 cm, Matrix 320 × 320, ETL 18, NEX 4, slice thickness 1 mm, no gap, number of slices 16, approximately 9 min duration. DTI was also performed and the parameters used were as follows: 16 directions of diffusion gradients, TR/TE 5700/92 ms, FOV 8 cm, Matrix 64 × 64, ETL 1, NEX 3, slice thickness 1 mm, no gap, number of slices 7, *b* = 800 s/mm^2^, approximately 4 min duration.

**Fig. 4:**
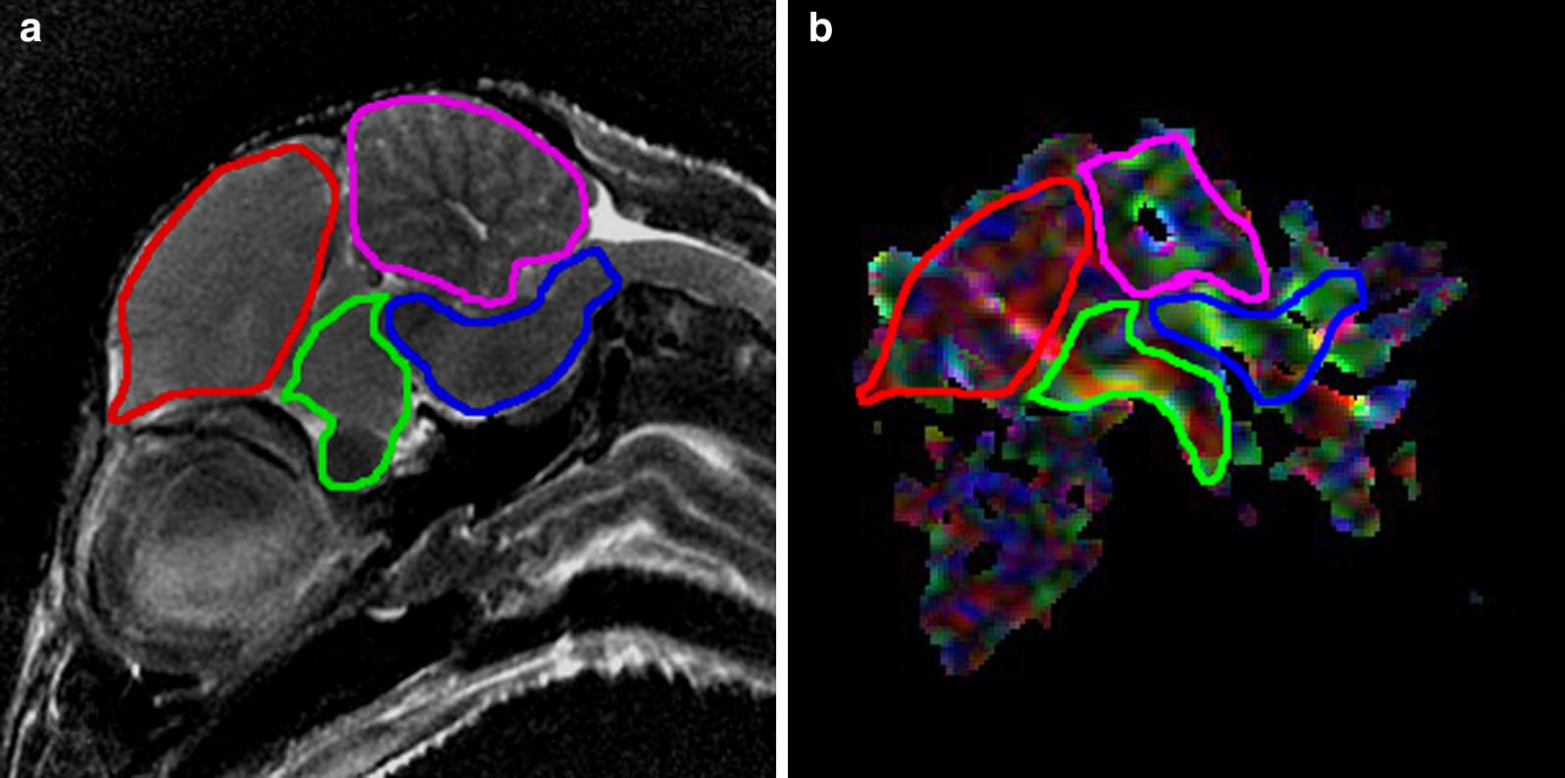
The segmentation result for different brain subdivisions in T2WI (**a**) and DTI (**b**). Segmented regions in T2WI were used as the regions of interest (ROIs) for DTI analysis. *Red outline* telencephalon; *pink outline* cerebellum; *green outline* optic lobe; *blue outline* brain stem

The areas of different parts of brain, such as the telencephalon, optic lobe, brain stem, and cerebellum, and the DTI parameters (ADC and FA value), were measured in the mid-sagittal slice for quantitative evaluation. Different anatomical regions, such as the telencephalon, optic lobe, brain stem, and cerebellum, were manually outlined in the images obtained by T2WI using the ImageJ software package (National Institutes of Health, Bethesda, MD). The area of the segmented region was then automatically calculated, and used as the quantitative measurement of brain size. Images obtained via DTI were analyzed using the built-in software in the GE workstation. Segmented regions of the telencephalon, optic lobe, brain stem, and cerebellum in the T2WI images were used as the regions of interest (ROIs) for DTI analysis. DTI parameters (ADC and FA value) were measured automatically after ROI placement. Figure 4 shows the brain segmentation result from T2WI and the ROI placement for DTI measurements from one chick in the radiation group.

### Statistical analysis

All data except the hatching and heteroplasia rates are expressed as mean ± standard deviation (SD). The statistical significance of the differences between the experimental and control groups was analyzed using the Chi-square test (for hatching rate and heteroplasia rate) and the student’s *t* test (for social behavior tests and MR-imaging measurements). The MedCalc software (Mariakerke, Belgium) was used for calculations. P < 0.05 was considered statistically significant.

## Results

### Effect on social behaviors of mobile phone radiation exposure during development

**Fig. 5:**
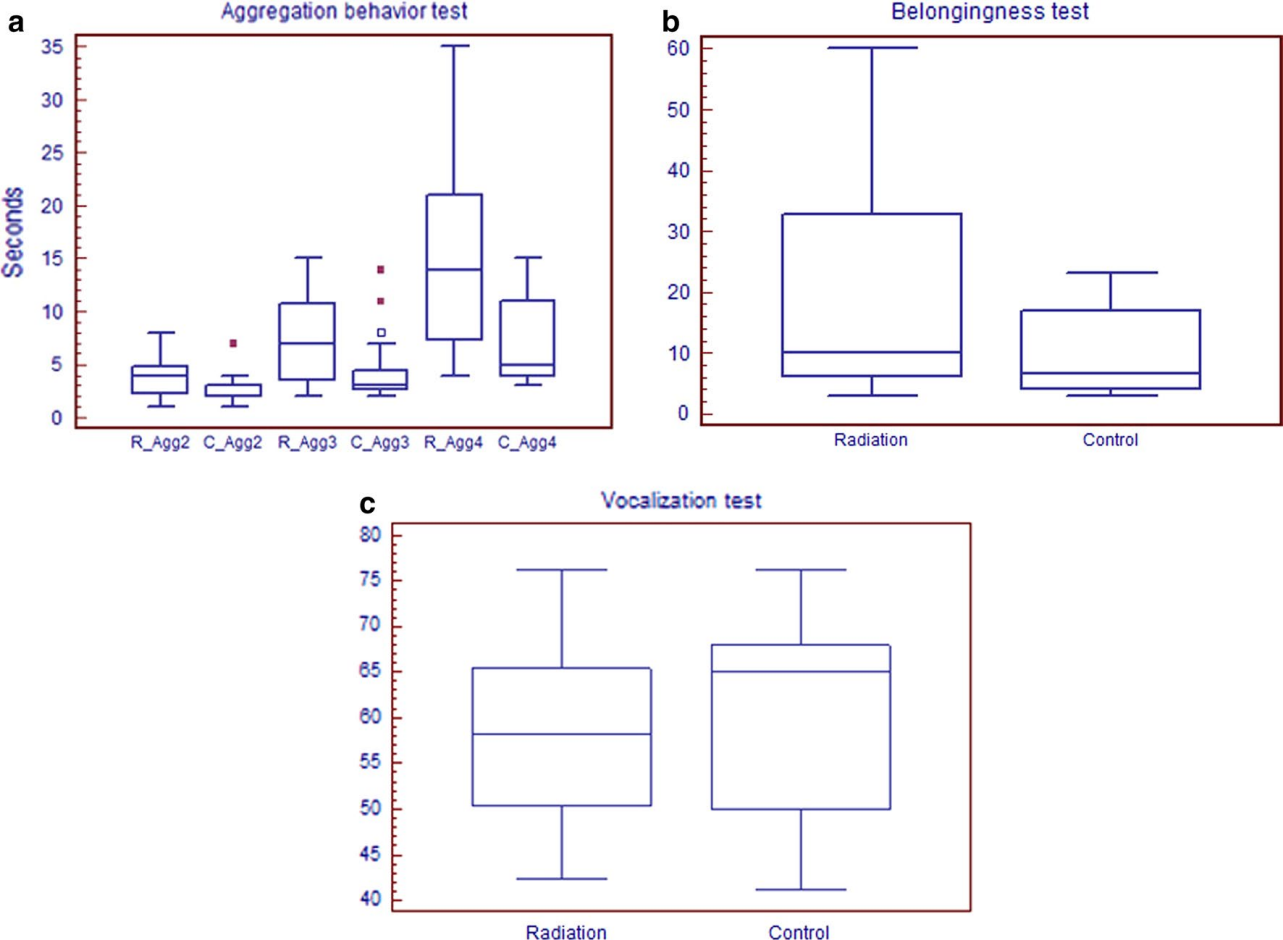
The results of social behavior tests. **a** Aggregation behavior test, **b** belongingness test, and **c** vocalization test. *R_Agg2*, *3*, *4*: the aggregation time of 2, 3, and 4 chicks in radiation group. *C_Agg2*, *3*, *4*: the aggregation time of 2, 3, and 4 chicks in control group

The results of the social behavior tests are shown in Fig. 5. In the aggregation behavior test, the duration of aggregation for three and four chicks was 7.07 ± 3.86 and 14.87 ± 10.06 s, respectively, in the radiation group; both of these were significantly longer than those of the control chicks (4.48 ± 3.16 s, P < 0.05; 7.48 ± 4.31 s, P < 0.05). The aggregation times for two chicks were not significantly different between the radiation and control groups (3.73 ± 1.79 vs. 2.81 ± 1.21 s, P > 0.05). In the belongingness test, the time taken to reach the goal from the isolated corner was 23.71 ± 8.72 s in the radiation group; this was significantly longer than that in the control group (11.45 ± 6.53 s, P < 0.05). In the vocalization test, the sound intensity of chicks in the radiation group was significantly weaker than that in control group (53.23 ± 8.60 vs. 60.01 ± 10.45 dB/30 s, P < 0.05).

### MRI findings on brain size and structural maturity

The size of different brain subdivisions, such as the telencephalon, optic lobe, cerebellum, and brain stem, and the corresponding DTI parameters (ADC and FA value) are shown in Table 2. No significant effect of mobile phone radiation exposure was found except on the size of the cerebellum (28.40 ± 1.95 cm^2^ in radiation group vs. 29.95 ± 1.41 cm^2^ in control group, P < 0.05).

**Table 2:** T2WI-based size estimate and DTI parameters (ADC and FA value) of different chick brain subdivisions (telencephalon, optic lobe, cerebellum, and brain stem) in radiation and control groups

Brain region	Area (cm^2^)		ADC 10^-3^ (mm^2^/s)		FA	
	Radiation	Control	Radiation	Control	Radiation	Control
Telencephalon	36.67 ± 1.75	37.29 ± 2.18	1.174 ± 0.370	1.343 ± 0.435	0.302 ± 0.048	0.353 ± 0.038
Optic lobe	15.68 ± 1.07	16.34 ± 1.09	1.125 ± 0.254	1.193 ± 0.200	0.243 ± 0.041	0.236 ± 0.033
Cerebellum	28.40 ± 1.95*	29.95 ± 1.41*	0.964 ± 0.251	0.837 ± 0.313	0.223 ± 0.040	0.219 ± 0.037
Brain stem	16.75 ± 1.13	16.89 ± 0.99	1.244 ± 0.284	1.403 ± 0.280	0.208 ± 0.039	0.212 ± 0.028

*DTI* diffused tensor imaging, *ADC* apparent diffusion coefficient, *FA* fractional anisotropy

* P < 0.05

### Hatching and heteroplasia rates

Table 3 shows the number of successfully hatched chicks from day 20 to 23 of incubation and the number of unhatched eggs in both the radiation and control group. Thirty chicks were successfully hatched in the radiation group, although four of them exhibited heteroplasia: one exhibited cyclopia and three were unable to stand. Twenty-eight chicks were successfully hatched in the control group, although two of them exhibited heteroplasia (unable to stand). Seven and eleven eggs were unhatched in the radiation and control group respectively. Chicks or embryos in these unhatched eggs were confirmed dead at day 25 of incubation. The hatching rate was not significantly different between the radiation and control groups (81.08 vs. 71.79 %, P > 0.05), and neither was the heteroplasia rate (10.81 vs. 5.12 %, P > 0.05).

**Table 3:** The number of chicks hatching from day 20 to 23 of incubation, the number of unhatched eggs, the hatching rate, and the heteroplasia rate in radiation (n = 37) and control group (n = 39)

	D 20	D 21	D 22	D 23	No. of unhatched eggs	No. of hatched chicks (hatching rate)	No. of hatched chicks with heteroplasia (heteroplasia rate)
Radiation (n = 37)	22	6	2	0	7	30 (81.08 %)	4 (10.81 %)
Control (n = 39)	20	8	0	0	11	28 (71.79 %)	2 (5.12 %)

## Discussion

We investigated the effect of mobile phone radiation exposure during embryogenesis on chick social behaviors, brain size, and the structural maturity of different brain subdivisions after hatching. The brain development of chicks is quite similar to that of humans [24, 25]. Firstly, the brains of both chicks and human newborns are well developed at hatching and birth, respectively. Secondly, the timing of brain development in chicks corresponds to that in human. Since the brains of rodent embryos mature quite late and are not completely developed at birth [26], the chick embryo is a more suitable animal model than the rodent to investigate the effect of exposure to environmental factors during embryogenesis on brain development.

Our present research established that exposure to mobile phone radiation during embryonic development has an adverse effect on chick social behaviors after hatching, reducing aggregation time and belongingness, and resulting in weaker vocalization. Similar animal studies have been carried out in rat offspring, yielding similar results [27, 28]. Here, T2-weighted MRI showed no adverse effect of radiation exposure on the size of different brain subdivisions, except the cerebellum. The cerebellum size in the radiation group was smaller than that in the control group. The cerebellum is associated with motor coordination and balance skills. The retardation of cerebellar development may influence these functions and manifest as an adverse effect on social behaviors. A previous electrophysiological and behavioral study showed that EMF radiation affects the cerebellar function of rat offspring [28]. EMF radiation emitted from mobile phones may cause structural damage to neurons. However, using 3.0 T DTI, no differences in structural maturity were observed in the different brain subdivisions examined. The relatively low spatial resolution of 3.0 T diffusion imaging (1.25 mm × 1.25 mm) may limit the discrimination of microstructural changes in certain brain regions, and micro-MRI with higher magnetic field strength could be used for further confirmation.

The mechanism underlying the effect of electromagnetic radiation on brain growth has not been fully clarified. Attention has widely been paid to the harmful effects of oxidative stress caused by electromagnetic radiation exposure during embryogenesis. Moderate oxidative stress promotes neuronal differentiation and proliferation; however, excessive oxidative stress causes apoptosis and necrosis [29]. The embryo is most sensitive to oxidative stress in the early developmental stage. With the development-related formation of antioxidant defenses, the embryo becomes more resistant to oxidative stress. The balance between moderate oxidative stress and the embryo’s antioxidant defenses is important for neuronal survival. “Reductive stress” caused by antioxidants may be as dangerous to neuronal survival as oxidative stress [30]. In addition, the magnitude of oxidative stress is different in different regions of brain. Electromagnetic radiation may upset the balance between the oxidative and anti-oxidative stress systems in a specific region of the brain during the period of brain growth and thus affect its function.

The effect of mobile phone radiation exposure on the chick hatching rate has been investigated in some studies, but results are conflicting [16, 19, 31]. The different sample sizes and exposure periods may have influenced the results. In the present study, we found no significant differences in the hatching and heteroplasia rates between the radiation and control groups. The sample size was too small to perform adequate statistical analysis of the hatching and heteroplasia rates. However, the effect of mobile phone radiation on chick hatching and heteroplasia rates was not the main aim of the present research.

This study has several limitations. We used a simple two-dimensional ROI to measure the size of the different brain subdivisions and no anatomical validation was conducted. However, three-dimensional volume measurements in images can result in significant measurement errors because of a partial volume effect. Moreover, the anatomical validation of the volume of different brain subdivisions is difficult to perform because of specimen damage during segmentation. To exclude unfertilized and undeveloped eggs, we began the radiation treatment from 4 days of incubation. Normal development of the chick embryos could be confirmed at day 4 of incubation through observing the capillary network by candling the eggs in the dark. However, the influence of mobile phone radiation during the very early developmental stage before day 4, when the neural tube begins to form, was not investigated in the present study. Different radiation exposure periods and the corresponding effects on social behavior test results, brain size, and structural maturity should be further explored and will be pursued in our future studies.

## Conclusions

In conclusion, mobile phone radiation exposure during chick embryogenesis impairs social behaviors after hatching and possibly delays cerebellar development, which indicates potentially adverse effects of mobile phone radiation on brain development.

## Abbreviations

ADC: apparent diffusion coefficient
DTI: diffusion tensor imaging
EMF: electromagnetic field
FA: fractional anisotropy
FSE: fast spin-echo
GSM: global system for mobile communication
MRI: magnetic resonance imaging
ROI: region of interest
SSST: social-separation-stress test

## References

[1] http://www.itu.int/en/ITU-D/Statistics/Documents/facts/ICTFactsFigures2015.pdf.

[2] Thomée S, Härenstam A, Hagberg M (2011). Mobile phone use and stress, sleep disturbances, and symptoms of depression among young adults—a prospective cohort study. BMC Public Health.

[3] Nathan N, Zeitzer J (2013). A survey study of the association between mobile phone use and daytime sleepiness in California high school students. BMC Public Health.

[4] Adams JA, Galloway TS, Mondal D, Esteves SC, Mathews F (2014). Effect of mobile telephones on sperm quality: a systematic review and meta-analysis. Environ Int.

[5] Coureau G, Bouvier G, Lebailly P, Fabbro-Peray P, Gruber A, Leffondre K, Guillamo JS, Loiseau H, Mathoulin-Pélissier S, Salamon R, Baldi I (2014). Mobile phone use and brain tumours in the CERENAT case–control study. Occup Environ Med.

[6] Gorpinchenko I, Nikitin O, Banyra O, Shulyak A (2014). The influence of direct mobile phone radiation on sperm quality. Cent European J Urol.

[7] Lagorio S, Röösli M (2014). Mobile phone use and risk of intracranial tumors: a consistency analysis. Bioelectromagnetics.

[8] Hossmann KA, Hermann DM (2003). Effects of electromagnetic radiation of mobile phones on the central nervous system. Bioelectromagnetics.

[9] Manikonda PK, Rajendra P, Devendranath D, Gunasekaran B, Channakeshava, Aradhya RS, Sashidhar RB, Subramanyam C (2007). Influence of extremely low frequency magnetic fields on Ca^2+^ signaling and NMDA receptor functions in rat hippocampus. Neurosci Lett.

[10] Valberg PA, van Deventer TE, Repacholi MH (2007). Workgroup report: base stations and wireless networks-radiofrequency (RF) exposures and health consequences. Environ Health Perspect.

[11] Blank M, Goodman R (2009). Electromagnetic fields stress living cells. Pathophysiology.

[12] Divan HA, Kheifets L, Obel C, Olsen J (2008). Prenatal and postnatal exposure to cell phone use and behavioral problems in children. Epidemiology.

[13] Guxens M, van Eijsden M, Vermeulen R, Loomans E, Vrijkotte TG, Komhout H, van Strien RT, Huss A (2013). Maternal cell phone and cordless phone use during pregnancy and behaviour problems in 5-year-old children. J Epidemiol Community Health.

[14] Finnie JW, Blumbergs PC, Cai Z, Manavis J, Kuchel TR (2006). Effect of mobile telephony on blood-brain barrier permeability in the fetal mouse brain. Pathology.

[15] Kumlin T, Iivonen H, Miettinen P, Juvonen A, van Groen T, Puranen L, Pitkäaho R, Juutilainen J, Tanila H (2007). Mobile phone radiation and the developing brain: behavioral and morphological effects in juvenile rats. Radiat Res.

[16] Batellier F, Couty I, Picard D, Brillard JP (2008). Effects of exposing chicken eggs to a cell phone in “call” position over the entire incubation period. Theriogenology.

[17] Ingole IV, Ghosh SK (2012). Effect of exposure to radio frequency radiation emitted by cell phone on the developing dorsal root ganglion of chick embryo: a light microscopic study. Nepal Med Coll J.

[18] Rashidi H, Sottile V (2009). The chick embryo: hatching a model for contemporary biomedical research. BioEssays.

[19] Tsybulin O, Sidorik E, Kyrylenko S, Henshel D, Yakymenko I (2012). GSM 900 MHz microwave radiation affects embryo development of Japanese quails. Electromagn Biol Med.

[20] Umur AS, Yaldiz C, Bursali A, Umur N, Kara B, Barutcuoglu M, Vatansever S, Selcuki D, Selcuki M (2013). Evaluation of the effects of mobile phones on the neural tube development of chick embryos. Turk Neurosurg.

[21] Nishigori H, Kagami K, Takahashi A, Tezuka Y, Sanbe A, Nishigori H (2013). Impaired social behavior in chicks exposed to sodium valproate during the last week of embryogenesis. Psychopharmacology.

[22] Haba G, Nishigori H, Sasaki M, Tohyama K, Kudo K, Matsumura Y, Sugiyama T, Kagami K, Tezuka Y, Sanbe A, Nishigori H (2014). Altered magnetic resonance images of brain and social behaviors of hatchling, and expression of thyroid hormone receptor βmRNA in cerebellum of embryos after methimazole administration. Psychopharmacology.

[23] Liu F, Garland M, Duan Y, Stark RI, Xu D, Bansal R, Dong Z, Peterson BS, Kangarlu A (2010). Techniques for in utero, longitudinal MRI of fetal brain development in baboons at 3T. Methods.

[24] Bernal J (2007). Thyroid hormone receptors in brain development and function. Nat Clin Pract Endocrinol Metab.

[25] Darras VM, Van Herck SL, Geysens S, Reyns GE (2009). Involvement of thyroid hormones in chicken embryonic brain development. Gen Comp Endocrinol.

[26] Rodier PM (1980). Chronology of neuron development: animal studies and their clinical implications. Dev Med Child Neurol.

[27] Jensh RP (1984). Studies of the teratogenic potential of exposure of rats to 6000-MHz microwave radiation. II. Postnatal psychophysiologic evaluations. Radiat Res.

[28] Haghani M, Shabani M, Moazzami K (2013). Maternal mobile phone exposure adversely affects the electrophysiological properties of Purkinje neurons in rat offspring. Neuroscience.

[29] Dennery PA (2007). Effects of oxidative stress on embryonic development. Birth Defects Res C Embryo Today.

[30] Castagné V, Lefèvre K, Natero R, Clarke PG, Bedker DA (1999). An optimal redox status for the survival of axotomized ganglion cells in the developing retina. Neuroscience.

[31] Saito K, Suzuki K, Motoyoshi S (1991). Lethal and teratogenic effects of long-term low-intensity radio frequency radiation at 428 MHz on developing chick embryo. Teratology.

